# Histopathological assessment of optic nerve invasion guided by radiological findings in enucleated globes with retinoblastoma

**DOI:** 10.1186/s12886-020-01654-z

**Published:** 2020-09-29

**Authors:** Mohammed M. Abusayf, Hind M. Alkatan, Sahar Elkhamary, Saleh A. Almesfer, Azza M. Y. Maktabi

**Affiliations:** 1 Department of Ophthalmology, College of Medicine, King Saud University, PO Box 18097, Riyadh, postal code 11415 Saudi Arabia; 2 Department of Pathology, College of Medicine, King Saud University Medical City, PO Box 18097, Riyadh, 11415 Saudi Arabia; 3 Radiology Department, King Khaled Eye Specialist Hospital, Riyadh, Saudi Arabia; 4 Diagnostic Radiology Department, Mansoura Faculty of Medicine, Mansoura, Egypt; 5 Pediatric Ophthalmology Division, King Khaled Eye Specialist Hospital, Riyadh, Saudi Arabia; 6 Pathology and Laboratory Medicine Department, King Khaled Eye Specialist Hospital, Riyadh, Saudi Arabia

**Keywords:** Retinoblastoma, High-risk, Histopathology, Optic nerve invasion, Lamina cribrosa, Radiology, Magnetic resonance imaging, Chemotherapy, Retrolaminar, Post-laminar

## Abstract

**Background:**

Optic nerve (ON) invasion is an important high-risk feature, and an indicator for neoadjuvant chemotherapy and prognosis. We aim through this study to correlate the detected-ON invasion by Magnetic resonance imaging (MRI) with the corresponding confirmed histopathological level of invasion.

**Methods:**

A retrospective study of enucleated globes with the diagnosis of retinoblastoma received in the histopathology department(s) from January 2015 to December 2016 (2 years). Slides were reviewed for ON invasion assessment, charts were reviewed for basic demographic data. All patients underwent MRI under sedation upon diagnosis and MRI findings were collected for the above correlation.

**Results:**

A total of 38 patients were included: 21 males and 17 females. 29 (77.3%) had unilateral involvement, 7 (18.4%) had bilateral involvement and 2 cases had trilateral disease. The overall mean age at diagnosis was 22.63 ± 15.15 months. Histopathological examination revealed ON invasion in 28 cases (74%) distributed as follows: prelaminar (31.6%), laminar (18.4%), and post-laminar (23.7%). MRI confirmed post-laminar ON invasion in 8 cases (true positive) but failed to detect this in 1 case. Additionally, MRI detected another 8 cases of ON invasion that were false positive on histopathology (accuracy: 63.3%; sensitivity: 88.9%; specificity: 72.4%; Positive predictive value (PPV): 50%; Negative predictive value (NPV): 95.5%).

**Conclusions:**

MRI is found to be less sensitive in evaluating prelaminar and laminar ON invasion (0.0 and 42.9%) compared to post-laminar invasion (88.9%). MRI has generally better specificity in detecting ON invasion irrespective of the invasion level. In our study, obtaining deeper and/or additional histologic sections from the other surface of the tissue block in cases where a post-laminar ON invasion by MRI is found but not confirmed histopathologically in routine sections is essential to avoid missing such an important high-risk feature.

## Background

Retinoblastoma (RB) is the commonest intraocular tumor in children representing approximately 4% of all pediatrics malignancy [1]. It can present as unilateral, bilateral or trilateral disease (bilateral RB tumors and pineoblastoma as a third intracranial tumor) [2].

The former Reese–Ellsworth classification, created in 1960s, was used to predict globe salvage with external beam radiotherapy when this used to be the most popular non-surgical treatment avoiding enucleation [3]. Recommendations were proposed to update this classification to include the current treatment modalities and outcome [4]. The International Classification of Retinoblastoma (ICRB) was introduced and finalized by a group of RB experts in April 2003, which is being used in this study [5]. The main goal of ICRB was being applicable to predict treatment success with current modalities such as chemo-reduction (CRD) therapy. This classification, is based on the number, location and size of RB tumor as well as the presence or absence of vitreous and subretinal seeds and whether they are localized or diffuse [5].

Management of RB in general is tailored to each individual patient, but several factors play important roles in each case including: metastatic risk, risk for second tumors, systemic condition, laterality, size and location of the tumor(s) and potential for vision. The priority is to detect and treat life-threatening conditions, then to save the globe and finally maintain vision [1]. Current management modalities include: intravenous chemo-reduction, intra-arterial chemotherapy, thermotherapy, cryotherapy, laser photocoagulation, plaque radiotherapy, external beam radiotherapy, and enucleation [1, 3, 6].

Uniform consensus as to what constitutes high-risk pathology has not been reached and high-risk pathological features have been described with few debates in the literature [7–9]. However, it has been agreed among many experts that the high-risk features should include: post-laminar optic nerve (ON) invasion, massive choroidal invasion, combined ON and choroidal invasion (of any type) or anterior segment invasion (infiltration of anterior uveal stroma) [9–11].

The presence of high-risk histopathological features after primary enucleation is an indication for adjuvant chemotherapy in view of increased risk of metastasis. Survival in these children increased significantly because of adjuvant chemotherapy [8]. On the other hand, secondarily enucleated globes following neoadjuvant chemotherapy did not seem to reduce the chance of harboring high-risk pathological features when compared to primarily enucleated globes [12]. In another study, invasion of the anterior structures (anterior chamber, iris, and ciliary body) was significantly more detected in secondarily enucleated globes with RB [13].

Based on the international RB staging work group for histopathological studying and globe preparation, ON invasion level has been classified as being prelaminar, laminar (intralaminar), post-laminar and involving surgical margin. Consensus on choroidal invasion has been also reached, where this can be focal or massive (massive choroidal invasion is defined as having diameter of 3 mm or more in any tumor dimension) [14]. Magnetic resonance imaging (MRI) is now becoming the most widely used modality in the workup for RB staging and assessment prior to primary enucleation [15]. To detect risk factors for metastasis, MRI is a helpful tool but not as reliable as histopathology, where microscopic infiltration is best detected [11]. Generally, the role of MRI in RB assessment includes: determination of the growth pattern, extension of the ON involvement, detection of orbital and/or meningeal extension, and the presence of second tumors [16]. Additionally, detection of ON invasion on MRI in children treated with primary enucleation might have a role in helping the surgeons to ensure free resection margin [17].

The aim of this study is to correlate the detected-ON invasion by imaging with the corresponding histopathological level of invasion. The cases where MRI showed more advanced level of ON invasion than what has been detected on histopathology were further subjected to more sectioning either by obtaining deeper levels or by sectioning the globes after rotating the blocks.

## Methods

This is an approved retrospective study on an expedited basis by the HEC/IRB at King Khaled Eye Specialist Hospital (KKESH) with collaborative agreement with King Saud University.

The histologic slides of all retinoblastoma enucleated globes received in the histopathology department(s) from January 2015 to December 2016 (2 years) were collected for review by 2 pathologists and the charts were reviewed for basic demographic data including: age at the time of study, age of presentation, gender, timing of enucleation, laterality, family history and previous treatment modalities if present. All patients underwent MRI under sedation upon diagnosis with ultrasonography.

MRI reading was done by an experienced neuroradiologist (SE). All patients underwent MRI of the orbit and brain with a 3.0-T system (Signa HDxt, GE Medical System) by using an eight-channel head coil under sedation. Sedation was achieved following the administration of oral chloral hydrate to infants and children who were less than 5-years of age. The conventional ocular MR imaging protocol comprised axial unenhanced T 1-weighted spin-echo images (TR/TE, 500/11 ms) and fat suppressed axial T 2-weighted images (TR/TE, 3450/90 ms). Pre- and post-contrast axial T1weighted MRIs with and without fat suppression were obtained. Contrast-enhanced fat suppression T1 weighted MRI after intravenous injection of 0.1 mmol kg21gadopentate dimeglumine was done in axial and coronal planes as well as the parasagittal plane parallel to the long axis of the optic nerve (TR/TE, 560/11 ms) after intravenous injection of Gd-DTPA (Magnevist; Bayer-Schering Pharma AG, Berlin, Germany) with 0.2 mL/ kg of body weight). High-resolution three-dimensional (3D) FIESTA (Fast Imaging Employing Steady-state Acquisition) allowed thin sections (0.4 mm) with high SNR sequence allowing performance of multiplanar reconstruction to better demonstrate tumor extension. Slice thickness was 3 mm, with an inter- section gap of 0.5 mm. The FOV was 18 cm with a matrix of 256 × 160. Additionally, images covered the whole brain including axial T 2-weighted images, as well as axial post-contrast T1-weighted images with a slice thickness of 5 mm, were obtained to check for intracranial lesions including pineal gland and suprasellar assessment (trilateral retinoblastoma) as well as abnormal meningeal enhancement. Diffusion-weighted MRI was acquired in the axial plane using a single-shot echo-planar imaging sequence (TR/TE, 8000/70s; slice thickness, 3 mm; intersection gap, 0.5 mm; FOV, 18 cm; and matrix, 128 × 128. excitation, 2). A b value of 0 and of 1000 s/mm2 was also applied in three orthogonal (z, y and x) directions. The time interval between MRI and enucleation operation was measured and varied from 1 to 128 days (Average of 4.0 days).

The histopathological results of the enucleated eyes utilizing the examination of the routine Hematoxylin and eosin (H&E) slides and a single Periodic Acid-Schiff (PAS) stained slide prepared from all 4 blocks: the pupil-optic nerve (PO) globe section, the 2 calottes (each one in a separate block) and the ON surgical margin of excision according to the protocol and guidelines of the Proceedings of the consensus meetings from the International Retinoblastoma Staging Working Group (IRSWG) on the pathology guidelines for the examination of enucleated eyes [14]. The histologic sections were reviewed for the following features: tumor differentiation and high-risk features including invasion of the optic nerve (none, prelaminar, laminar, or post-laminar, transection end). The enucleated globes reviewed over the study period were filtered to extract the cases where the level of ON invasion histopathologically (based on the IRSWG Consensus) did not match the corresponding pre-operative MRI reported level. The cases in which MRI studies showed deeper ON level of invasion than what was documented in the initial histopathology examination where re-evaluated by further deeper serial sections from the PO tissue block. In some cases, flipping of the PO block was tried to re-examine the ON from the other side. The additional histopathologic sections were further evaluated as a second stage of the study.

We also determined the accuracy, sensitivity, specificity, positive predictive value (PPV) and negative predictive value (NPV) of MRI in predicting ON tumor invasion.

All data was entered in an excel sheet (Microsoft office excel 2011 for Mac). Statistical analysis was performed with SPSS version for Microsoft Windows (SPSS, Inc., Chicago, IL, USA). Sensitivity was calculated as a percentage by dividing true-positive findings by the sum of true-positive and false-negative findings. Specificity was calculated as a percentage by dividing true-negative findings by the sum of true-negative and false-positive findings. Accuracy was also calculated.

## Results

A total of 44 patients were initially identified during the study period, out of which, 6 patients were excluded because of pre-enucleation treatment. A total of 38 patients were included: 21 males and 17 females. Out of these patients, 29 (77.3%) had unilateral involvement, 7 (18.4%) had bilateral involvement and 2 cases had trilateral disease. The overall mean age at diagnosis was 22.63 ± 15.15 months. (Table 1).

**Table 1:** Descriptive analysis of our included retinoblastoma cases (*n* = 38)

Characteristic	N (%)
Gender	
Male	21 (55.3)
Female	17 (44.7)
Family history	
Positive	2 (5.3)
Negative	36 (94.7)
Laterality	
Unilateral	29 (76.3)
Bilateral	7 (18.4)
Trilateral	2 (5.3)
Studied eye	
Right	19 (50.0)
Left	19 (50.0)
Age at presentation (months)	
Overall (n = 38) mean ± SD [Range]	22.6 ± 15.2 [2-72]
Unilateral (*n* = 29) mean ± SD [Range]	26.6 ± 15.0 [2–72]
Bilateral (n = 7) mean ± SD [Range]	8.0 ± 5.1 [2–17]
Trilateral (n = 2) mean ± SD [Range]	17.0 ± 1.4 [16-18]
Age at enucleation (months)	
Overall (n = 38) mean ± SD [Range]	22.9 ± 15.3 [2–72]
Unilateral (*n* = 29) mean ± SD [Range]	26.9 ± 15.2 [2–72]
Bilateral (*n* = 7) mean ± SD [Range]	8.1 ± 5.0 [2–17]
Trilateral (n = 2) mean ± SD [Range]	17.0 ± 1.4 [16–18]
Radiological evidence	
None	8 (21.1)
Pre-laminar	6 (15.8)
Laminar	8 (21.1)
Post-laminar	16 (42.1)
Histopathological evidence	
None	10 (26.3)
Pre-laminar	12 (31.6)
Laminar	7 (18.4)
Post-laminar	9 (23.7)
Radiological-Histopathological agreement	
Equal	15 (39.4)
Radiological < Histopathological	5 (13.2)
Radiological > Histopathological	18 (47.4)
Final decision for the Radiological > Histopathological (*n* = 18)	
No change	11 (61.1)
Pre-laminar	1 (5.6)
Laminar	1 (5.6)
Post-laminar	5 (27.8)
Histopathological evidence of choroidal invasion	
None	20 (52.6)
Focal	6 (15.8)
Massive	12 (31.6)
Diagnostic Ultrasonography findings	
No calcification	3 (7.9)
Calcification	35 (92.1)

Histopathological examination showed ON invasion in 28 eyes (74%) as follows: 12 eyes had prelaminar invasion (31.6%), 7 eyes had laminar invasion (18.4%), and 9 (23.7%) had post-laminar invasion (including 1 patient with surgical margin involvement).

**Fig. 1:**
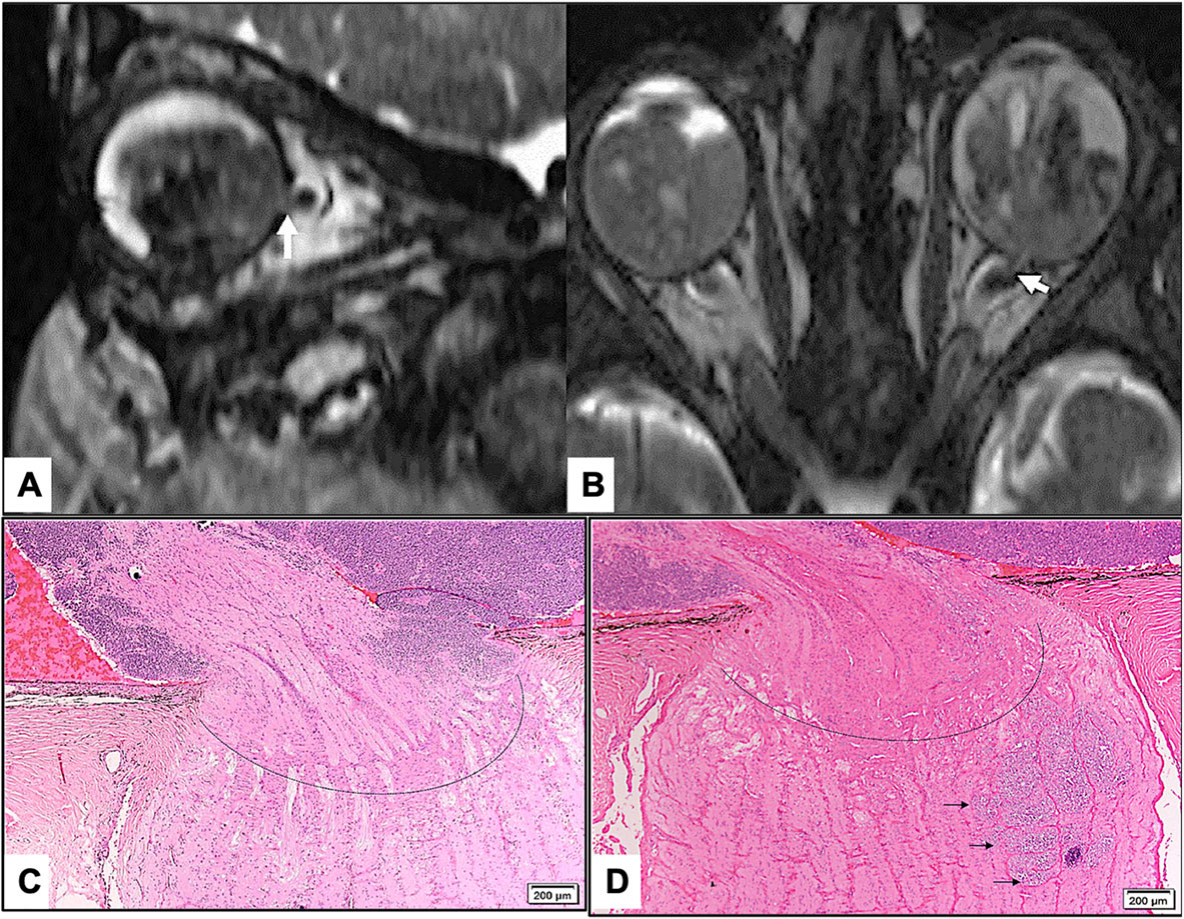
**a** and **b** High resolution Sagittal (**a**) and Axial T1 orbital Constructive Interference in Steady State (CISS) magnetic resonance imaging (**b**) prior to enucleation of left globe in a case with bilateral retinoblastoma showing a tumor mass with hemorrhage filling the posterior cavity, pushing the lens anteriorly, with optic nerve invasion beyond the lamina cribrosa. **c** and **d** Early Histopathological appearance of the optic nerve (ON) in the early initially submitted routine sections with tumor cells level of ON invasion (Dotted curve line) anterior to the lamina cribrosa (**c**) while deeper sections obtained were showing islands of tumor cells (black arrows) invading the ON posterior to the lamina cribrosa (**d**) (Original magnification × 200 Hematoxylin and eosin)

**Fig. 2:**
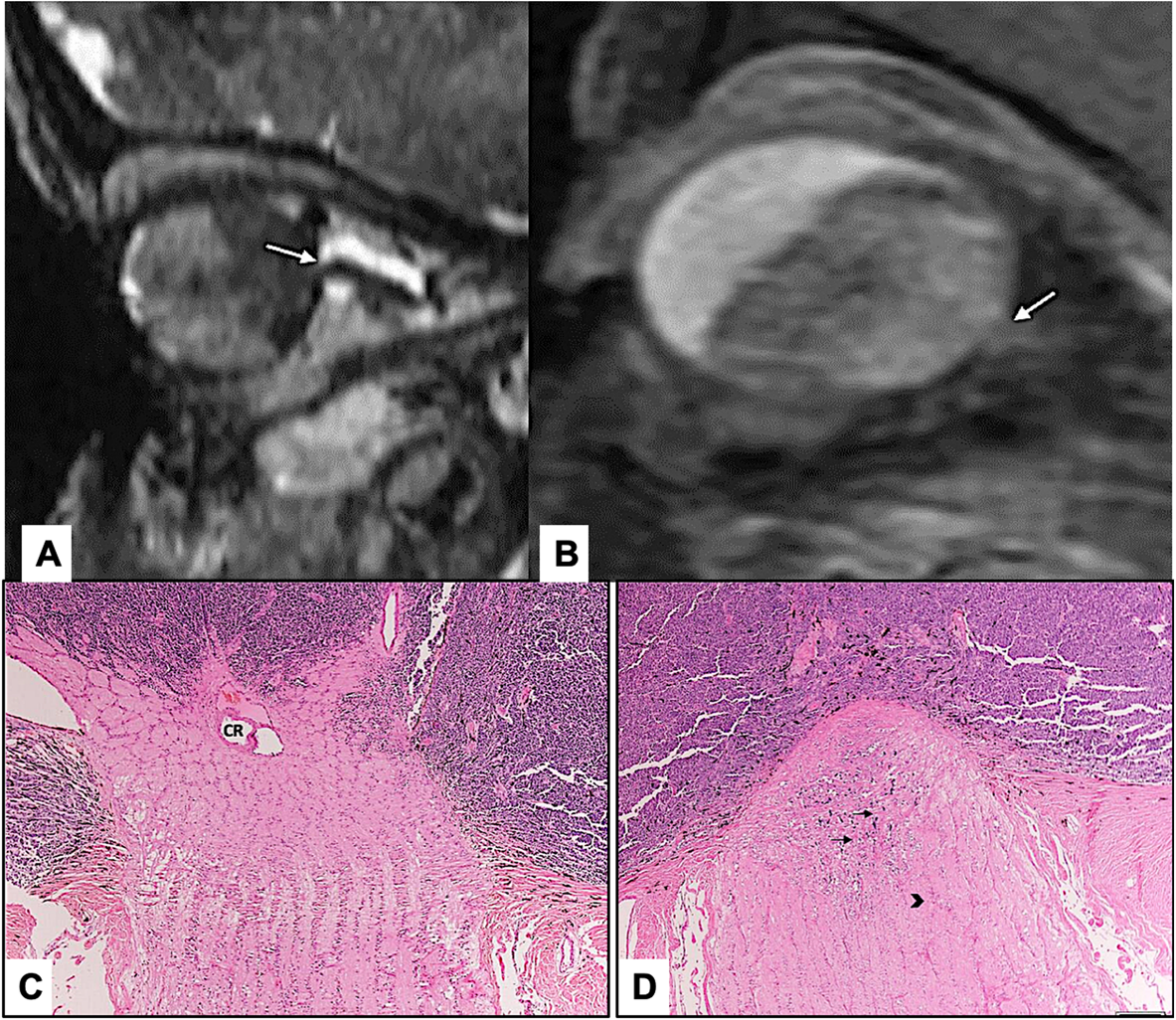
**a** and **b** High resolution Sagittal and Axial orbital Constructive Interference in Steady State (CISS) and post contrast fat suppressed magnetic resonance imaging (**a**) showing invasion of the optic nerve (ON) up to 3 mm post lamina cribrosa (white arrow). Sagittal oblique magnetic resonance imaging view of the orbit (**b**) showing the posterior cavity is almost totally filled by tumor with interruption of the choroidal-retinal interface line (white arrow). **c** and **d** Deeper histopathological sections of the ON at the level of central artery (indicated by CR) with superficial invasion by retinoblastoma (RB) tumor cells (**c**). Histological sections (**d**) obtained after flipping of the tissue block showing tumor cells invading the ON (black arrows) and extending posterior to the lamina cribrosa (arrow head) (Original magnification × 200 Hematoxylin and eosin)

**Fig. 3:**
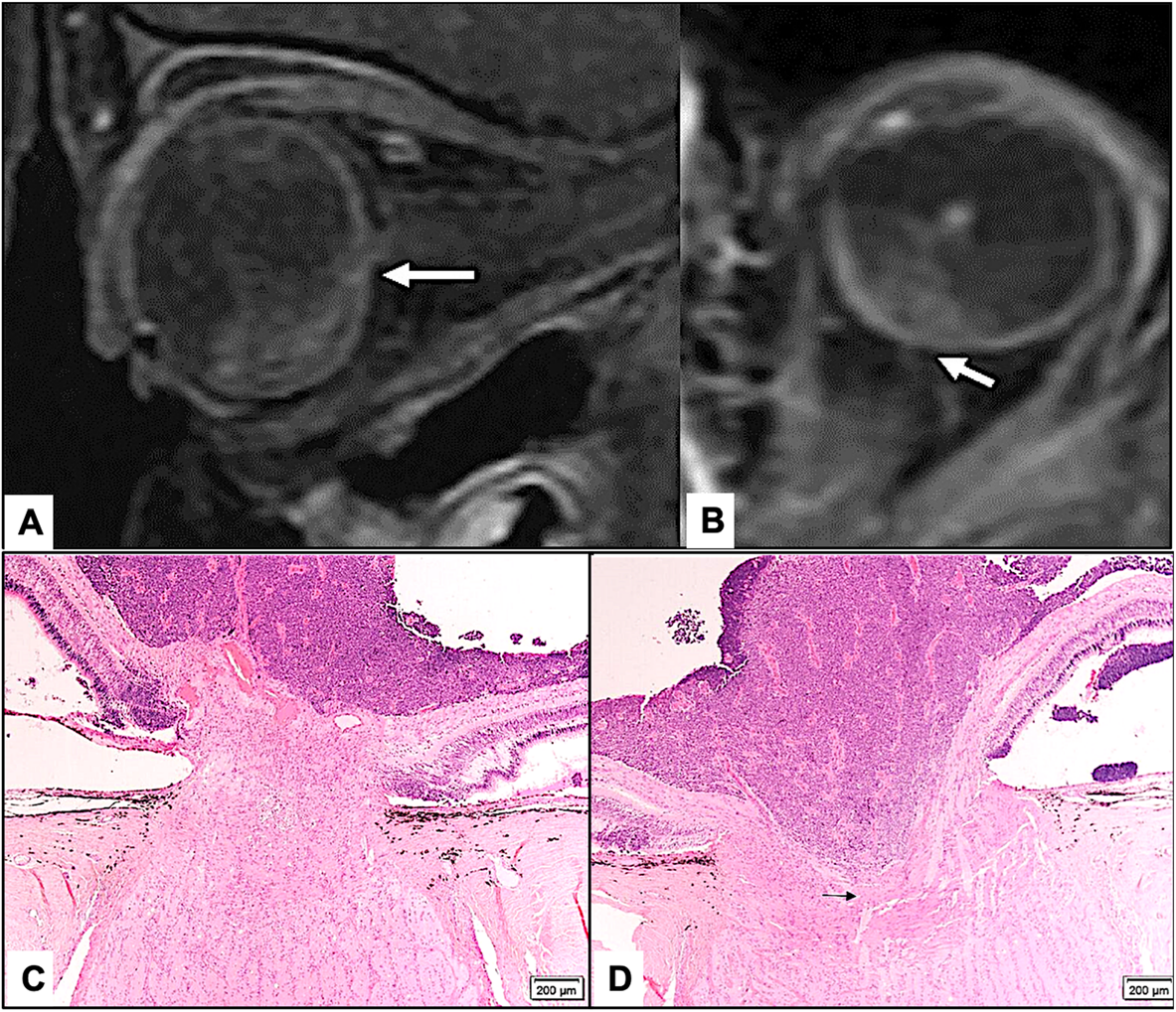
**a** and **b** Post-contrast fat suppressed Sagittal oblique and Axial magnetic resonance imaging of the orbit showing tumor with interruption of the choroidal-retinal interface line in (**a**) and optic nerve (ON) invasion of the lamina cribrosa extending for 1.5 mm within the ON beyond the lamina (**b**) (white arrows). **c** and **d** Initial histological sections at the level of central artery (CR) with No ON invasion detected in (**c**) while further deeper sections have shown tumor cells (black arrow) filling the glaucomatous ON cup and reaching to the lamina cribrosa (Original magnification × 200 Hematoxylin and eosin)

MRI showed ON invasion in 30 eyes (78%) as follows: pre-laminar in 6 eyes (15.8%), laminar in 8 eyes (21.1%), and post-laminar (including 1 patient with surgical margin involvement) in 16 (42.1%). There was an agreement between both modalities in 15 eyes (39.5%) with identical level of ON invasion, while disagreement was present in 23 eyes (60.5%). Less depth of invasion histopathologically was observed in 18 eyes (47.4%). Further histopathological sectioning in the second stage of the study resulted in reaching an agreement with the MRI finding of deeper ON invasion in 7/18 (40%) eyes. However, the level of ON invasion was not altered in remaining 11/18 (61.1%) eyes (Figs. 1, 2 and 3).

Post-laminar ON invasion was correctly identified in 8 cases (true positive), while another 8 were false positive. Only 1 case was missed on MRI (accuracy: 63.3%; sensitivity: 88.9%; specificity: 72.4%; Positive predictive value (PPV): 50%; Negative predictive value (NPV): 95.5%). MRI was found to be less sensitive in evaluating prelaminar and laminar ON invasion (0.0 and 42.9%) compared to post-laminar invasion (88.9%) but had better overall specificity in detecting invasion (72.4 to 83.9%). (Table 2).

**Table 2:** Sensitivity, specificity, accuracy, PPV and NPV of MRI compared to histopathology detection of ON invasion level (n = 38) excluding 6 cases that had treatment before enucleation

Optic nerve invasion	TP (n)	FP (n)	TN (n)	FN (n)	Accuracy (%)	Sensitivity (%)	Specificity (%)	PPV (%)	NPV (%)
None	4	4	24	6	67.2	40.0	85.7	50.0	80.0
Prelaminar	0	6	20	12	52.6	0.0	76.9	0.0	62.5
Laminar	3	5	26	4	71.4	42.9	83.9	37.5	86.7
Postlaminar	8	8	21	1	63.3	88.9	72.4	50.0	95.5

*TP* Total positive, *FP* False positive, *TN* Total negative, *FN* False negative, *PPV* Positive predictive value, *NPV* Negative predictive value

## Discussion

Many studies have been published correlating the clinical and MRI features in relation to RB high risk features [7, 14, 15]. In the era of targeted therapy like intra-arterial chemotherapy, identification of these features was important because many eyes with advanced disease were salvaged with non-surgical treatment [6]. The combined clinical and MRI features could predict high-risk RB, while ICRB and/or Reese-Ellworth classifications alone provided limited correlation with high-risk RB [10].

MRI works without the use of ionizing radiation, preventing the occurrence of the potential risk of secondary tumors and has a high soft tissue contrast [2]. Using special surface coils with small diameter and a short penetration depth improved the ability to correlate the MRI findings with the actual ocular histopathological findings [2, 18]. Results from many studies showed that preoperative MRI was more relevant in detecting ON invasion [17]. Others have shown different sensitivity and specificity of MRI in detecting the level of ON invasion [19]. Moreover, some investigators advocated neoadjuvant chemotherapy before enucleation in cases with extensive unilateral disease based on MRI detection of post-laminar ON invasion [11, 20, 21]. However, the decision for neoadjuvant chemotherapy based on MRI alone with no histopathological confirmation was not justified [11].

In 2007, Lemke in a prospective clinical trial showed that MRI sensitivity for the detection of ON infiltrations (prelaminar and post-laminar together) was 53.8% and the specificity was 82.3%. ON infiltrations were correctly recognized in seven out of 10 cases (true positive) and excluded in 14 out of 20 cases (true negative). The histopathological findings showed six cases of prelaminar ON infiltration with false negative results on MRI. Three cases were incorrectly diagnosed as prelaminar infiltration in MRI. Importantly, no post-laminar ON infiltration was missed. This might suggest a better ability of MRI to diagnose post-laminar ON invasion [2, 22]. Brisse found that MRI sensitivity in detecting post-laminar invasion in normal-size ONs is 60%, which was comparable to previous studies [17]. Sensitivities were significantly variable for post-laminar ON invasion. However, the specificity (true negative rate) and negative predictive values (NPV) were 87 and 93% respectively. Wilson in 2009, found limited correlation between MRI and histopathologic assessment of ON invasion in eyes with RB [19]. Furthermore, Chawla in 2012 concluded that the decision for neoadjuvant chemotherapy on the basis of suspected post-laminar invasion by MRI alone was not justified in the absence of histopathologic evidence because MRI had limitations in reliably predicting the microscopic infiltration of the choroid and optic nerve [11].

More recently, in 2018 Cui retrospectively reviewed 63 primary enucleated eyes with advanced RB that showed ON invasion by histopathological examination in 26 cases (41%). MRI studies failed to predict prelaminar and laminar ON invasion, thus indicating a low sensitivity and positive predictive value (PPV) of 42.9 and 37.5% for prelaminar invasion, 50.0 and 40.0% for laminar invasion, respectively). However, in the same paper, post-laminar ON invasion was diagnosed by MRI in 16 cases (25.4%), out of which 11 eyes (17.5%) were true positive and 5 (7.9%) were false positive. Only 4 cases were missed on MRI (accuracy: 85.7%; sensitivity: 73.3%; specificity: 89.6%; PPV: 68.8%; NPV: 91.5%) [23]. Li evaluated the value of MRI as a useful diagnostic tool for post-laminar optic nerve invasion with a calculated sensitivity of 82%, specificity of 73% and accuracy of 77% [24]. Similarly, in our series, ON invasion was found in 28 cases (74%) by histopathological examination, out of which 12 eyes had prelaminar invasion (31.6%), 7 eyes had laminar invasion (18.4%), and 9 (23.7%) had post-laminar invasion (including 1 patient with surgical margin involvement). ON invasion was evident by MRI in 30 (78%) patients distributed as follows: 6 (15.8%) eyes pre-laminar, 8 (21.1%) laminar, and 16 (42.1%) post-laminar (including 1 patient with surgical margin involvement). Post-laminar optic nerve invasion was correctly identified in 8 cases (true positive), while 8 cases were considered false positive. Only 1 case was missed by MRI study (sensitivity: 88.9%; specificity: 72.4%; accuracy: 63.3%; PPV: 50%; and NPV: 95.5%). In our series, MRI did not also predict ON invasion reliably and accurately in cases of prelaminar and laminar ON invasion, which was similar to previously published findings [25]. The histopathological confirmation of the level of ON invasion remains the gold standard for treatment planning. On the other hand, MRI proved to be useful in predicting post-laminar ON invasion with a better sensitivity, specificity and accuracy. Therefore, routine histopathological sections showing less depth of ON invasion in tissue than what was documented radiologically should be carefully re-evaluated with additional sections in order to reach the best possible agreement between these 2 diagnostic modalities. If the initial histopathological sections submitted do not reach the central part of the ON, the level of invasion can be underestimated, in which case deeper sections would be helpful. Also, the presence of ON invasion along one side of the ON rather than being equally present in a circumferential fashion around the ON central vessels may also result in missing the actual true area of invasion therefore obtaining sections from the other side of the tissue block (flipping) would confirm the invasion.

## Conclusions

MRI is a valuable tool in detecting ON invasion in enucleated globes with RB and has shown better specificity, sensitivity and accuracy in detecting post-laminar ON invasion. However, this is the first study demonstrating that disagreement between histopathological findings observed in routinely submitted sections of RB enucleated globes and the MRI findings may be encountered especially if no standardized protocols are universally followed in ophthalmic histopathology units across the world. The above disagreement rate can be reduced by obtaining further deeper and/or modified histopathological sections (with 180 degrees rotation of the tissue block). The MRI finding of a post-laminar ON invasion that is missed by histopathological examination in such cases is extremely important and should not be overlooked to avoid misleading assessment of this high-risk histopathological feature. Therefore, we strongly recommend an internationally used protocol in the histopathological assessment of enucleated globes with RB taking into consideration the radiological MRI findings with special attention in cases when post-laminar ON invasion by MRI fails to be proven by tissue diagnosis.

## Data Availability

The data for this study is stored at the institution where HEC/IRB approval has been obtained and can be made available upon request by the journal from the corresponding author otherwise the necessary data is shown in this manuscript.

## Abbreviations

RB: Retinoblastoma
ON: Optic nerve
MRI: Magnetic resonance imaging
ICRB: International Classification of Retinoblastoma
CRD: Chemo-reduction
IRSWG: International Retinoblastoma Staging Working Group
PO: Pupil-Optic nerve
NPV: Negative predictive value
PPV: Positive predictive value
